# Structural mechanism for regulation of the AAA-ATPases RUVBL1-RUVBL2 in the R2TP co-chaperone revealed by cryo-EM

**DOI:** 10.1126/sciadv.aaw1616

**Published:** 2019-05-01

**Authors:** Hugo Muñoz-Hernández, Mohinder Pal, Carlos F. Rodríguez, Rafael Fernandez-Leiro, Chrisostomos Prodromou, Laurence H. Pearl, Oscar Llorca

**Affiliations:** 1 Structural Biology Programme, Spanish National Cancer Research Centre (CNIO), Calle de Melchor Fernández Almagro 3, 28029 Madrid, Spain.; 2 Genome Damage and Stability Centre, School of Life Sciences, University of Sussex, Falmer, Brighton, UK.

## Abstract

The human R2TP complex (RUVBL1-RUVBL2-RPAP3-PIH1D1) is an HSP90 co-chaperone required for the maturation of several essential multiprotein complexes, including RNA polymerase II, small nucleolar ribonucleoproteins, and PIKK complexes such as mTORC1 and ATR-ATRIP. RUVBL1-RUVBL2 AAA-ATPases are also primary components of other essential complexes such as INO80 and Tip60 remodelers. Despite recent efforts, the molecular mechanisms regulating RUVBL1-RUVBL2 in these complexes remain elusive. Here, we report cryo-EM structures of R2TP and show how access to the nucleotide-binding site of RUVBL2 is coupled to binding of the client recruitment component of R2TP (PIH1D1) to its DII domain. This interaction induces conformational rearrangements that lead to the destabilization of an N-terminal segment of RUVBL2 that acts as a gatekeeper to nucleotide exchange. This mechanism couples protein-induced motions of the DII domains with accessibility of the nucleotide-binding site in RUVBL1-RUVBL2, and it is likely a general mechanism shared with other RUVBL1-RUVBL2–containing complexes.

## Teaser

Cryo-EM reveals the remodeling of RUVBL1-RUVBL2 ATPases by the client recruitment component in the HSP90 co-chaperone R2TP.

## INTRODUCTION

R2TP (named after its components in yeast, Rvb1-Rvb2, Tah1 and Pih1) is an Heat shock protein 90 (HSP90) co-chaperone complex assembled from RuvB-like protein 1 (RUVBL1), RuvB-like protein 2 (RUVBL2), RNA polymerase–associated protein 3 (RPAP3), and PIH1 domain-containing protein 1 (PIH1D1) (*1*–*5*). RUVBL1 (Rvb1p in yeast) and RUVBL2 (Rvb2p in yeast) are essential and related AAA adenosine triphosphatases (ATPases) (*6*), while PIH1D1 (Pih1p in yeast) is a client recruitment protein recognizing casein kinase 2 (CK2)–phosphorylated adaptors containing a consensus DpSDD/E motif (*7*, *8*). RPAP3 contains two tetratricopeptide repeat (TPR) domains that recruit the HSP90 and HSP70 chaperone systems (*9*–*11*)—this function is fulfilled by the much smaller Tah1p protein in yeast. These chaperones, together with R2TP, facilitate the assembly and cellular stability of several macromolecular complexes, including complexes of the phosphatidylinositol 3-kinase–like family of kinases (PIKKs) such as mammalian Target of Rapamycin (mTOR) complex 1 (mTORC1), ATM and RAD3-related (ATR)-ATR-interacting protein (ATRIP), and Suppressor with morphogenetic effect on *genitalia* (SMG1) (*2*, *4*, *5*, *8*); small nucleolar ribonucleoproteins (*1*); RNA polymerase II (*9*, *10*); and possibly other multiprotein complexes (*3*, *12*).

R2TP associates with additional proteins in the cell, including prefoldin and prefoldin-like proteins that assemble the so-called R2TP/Prefoldin-like complex (*3*, *10*). In addition, RUVBL1 and RUVBL2 can engage with PIH1D1-like and RPAP3-like proteins to form several R2TP-like complexes (*12*, *13*). These may have specific functions or deal with specific clients during HSP90-mediated protein complex assembly, and/or they might be tissue specific. Evidence suggests a modular model where components of the R2TP/PFLD could associate differentially. Because of this complexity, the term PAQosome (particle for arrangement of quaternary structure) has been proposed to rename R2TP/PFDL (*14*). In addition to their function in R2TP and its variants, RUVBL1/Rvb1 and RUVBL2/Rvb2 are essential components of other complexes, such as the INO80 and SWR1 chromatin remodelers (*15*, *16*).

**Fig. 1:**
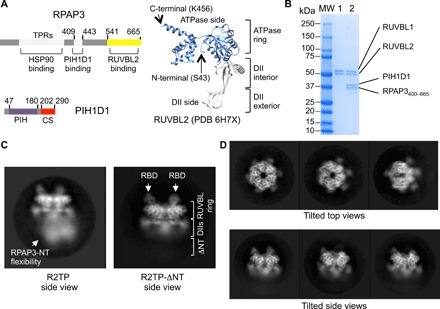
Purification and cryo-EM of R2TP-ΔNT. (**A**) Left: Components of R2TP and their domains. TPR, PIH, and CS domains are indicated. Right: Domains of a RUVBL2 subunit (PDB 6H7X, residues S43 to K456) (*26*), indicating the ATPase and DII side of the ring. (**B**) SDS-polyacrylamide gel electrophoresis for the final purification steps of RUVBL1-RUVBL2 (lane 1), which we mixed with the RPAP3_400–665_-PIH1D1 (lane 2) subcomplex. We used the sample in lane 2 for vitrification and cryo-EM. MW, molecular weight markers. (**C**) Representative side view 2D average of R2TP (*17*) and the truncated version produced in this work. Flexible regions in R2TP disappear in R2TP-ΔNT after the first 399 residues of RPAP3 are removed. RBD domains are labeled with white arrows, and the location of the RUVBL ring and DII domains is indicated. Images are at a different scale. (**D**) In the micrographs, multiple orientations of top, tilted, and side views were found, assuring a good coverage of Euler angles during image processing.

AAA ATPases RUVBL1 and RUVBL2 are the only common components in all the R2TP-like complexes found so far (*13*, *14*, *17*, *18*). RUVBL1 and RUVBL2 assemble into heterohexameric rings with domain II (DII) from each subunit protruding from one face of the ring, while the other is defined by the ATPase domains (Fig. 1A) (*19*–*23*). DII domains are composed of two segments, a flexible oligonucleotide/oligosaccharide-binding domain (DII external) and an internal region in proximity to the ATPase ring (DII internal). In yeast R2TP, the DII face of Rvb1p/Rvb2p interacts with the Pih1p-Tah1p proteins involved in client and Hsp90 recruitment, respectively (*24*, *25*). In human R2TP, PIH1D1 and the central and N-terminal regions of RPAP3 are also located at the DII face of the ring, but with a RUVBL2-binding domain (RBD) at the C-terminal end of RPAP3 stretching around the edge of the ring, to bind to the ATPase face of each RUVBL2 subunit. This interaction provides an anchor that permits substantial flexibility of the N-terminal regions of RPAP3 that mediate HSP90 and HSP70 binding (*17*). The N-termini of RUVBL1 and RUVBL2 both contain a long segment that is only partially resolved in crystal structures (Fig. 1A) (*20*, *22*, *26*).

PIH1D1 contains an N-terminal phosphopeptide-binding PIH domain that binds DpSDD/E motifs phosphorylated by CK2, which are present in a number of clients, such as Meiotic recombination 11 (MRE11), and adaptors, such as Telomere Maintenance 2 (TELO2) (*7*, *8*, *27*, *28*). TELO2 itself interacts with the TELO2-interacting protein 1 (TTI1) and TELO2-interacting protein 2 (TTI2) proteins to form the TTT complex, which bridges R2TP to PIKKs such as mTOR, SMG1, and ATR (*2*, *5*, *29*). PIH1D1 also contains a C-terminal CS domain consisting of seven β strands that bind to a long unstructured region in RPAP3 that loops around the CHORD domain-containing protein and Sgt1 (CS) domain (residues 409 to 443 of RPAP3 in the crystal structure of RPAP3_281–445_:PIH1D1_199–290_) (*11*). Constructs spanning residues 400 to 420 in RPAP3 are sufficient to pull down RPAP3, showing that this small region in RPAP3 is sufficient to form a stable complex with PIH1D1 (*17*). The structure of the RPAP3_281–445_:PIH1D1_199–290_ complex showed that a hydrophobic patch in the CS domain binds helices of the second TPR domain in RPAP3 (residues 281 to 396), thus contributing to the interaction between PIH1D1 and RPAP3 (*11*). Although RPAP3 and PIH1D1 form a clear 1:1 complex, the stoichiometry of these proteins in the full R2TP complex is not defined. Cryo–electron microscopy (cryo-EM) of human R2TP complexes shows the presence of up to three well-resolved copies of the C-terminal RPAP3-RBD domain bound to RUVBL2 on the ATPase face of the ring, suggesting that one, two, or three RPAP3 molecules may be bound in the R2TP complex (*17*). However, the stoichiometry of PIH1D1 could not be unambiguously defined through cryo-EM image processing owing to the variation in RPAP3 stoichiometry and the considerable conformational flexibility of the N-terminal regions of RPAP3, which map to the same face of the ring as PIH1D1.

The ability of the RUVBL1-RUVBL2 complex to bind and/or hydrolyze nucleotides is required for its documented cellular functions (*16*, *30*, *31*), and isolated RUVBL1-RUVBL2 in vitro displays a modest ATPase activity (*22*, *32*). However, the biochemical role of this nucleotide binding and ATPase activity in those functions and how and/or whether this activity is regulated are largely unknown. Access to the nucleotide-binding sites of both subunits is substantially restricted within all of the various heterohexameric RUVBL1-RUVBL2 ring structures that have been experimentally determined across multiple species (*20*, *22*, *32*). Thus, as with ATPases, such as HSP70 where the nucleotide-binding site also becomes occluded, efficient ATPase activity of RUVBL1-RUVBL2 may depend on interaction with another protein (or proteins) to switch the conformation of the ring and facilitate nucleotide exchange.

We have now eliminated the conformational heterogeneity of the N-terminal region of RPAP3, which confounded the structural analysis of PIH1D1 in previous cryo-EM studies of human R2TP, allowing unambiguous identification and localization of a single PIH1D1 bound to RUVBL2 on the DII face of the RUVBL1-RUVBL2 ring. We show that binding of PIH1D1 breaks the threefold symmetry of the ring and elicits a change in the conformation of the interacting RUVBL2-DII domain that is coupled to remodeling of the nucleotide-binding site to an open and more accessible state. These results identify PIH1D1 as a nucleotide exchange factor for RUVBL1-RUVBL2 within the R2TP complex and provide a model for the regulation of the inherent ATPase activity of RUVBL1-RUVBL2 that is likely to be common to other complexes in which it participates.

## RESULTS

### Reconstitution and cryo-EM of a truncated R2TP

RPAP3 (isoform 1) comprises segments that interact with HSP90 (residues 133 to 255 for TPR1 and residues 281 to 396 for TPR2), PIH1D1 (residues 409 to 443), and RUVBL2 (residues 541 to 665 for RBD) (Fig. 1A) (*11*, *17*). As flexibility and consequent conformational heterogeneity in the N-terminal regions adversely affected cryo-EM image processing in previous studies (*17*), we sought to improve structural resolution of the PIH1D1 component of human R2TP by removing the TPR domain regions from RPAP3 (*17*). We purified RUVBL1-RUVBL2 (R2) and RPAP3-PIH1D1 (TP) subcomplexes independently, using an RPAP3 construct (RPAP3_400–665_) devoid of the TPR domains and extreme N terminus but retaining the ability to bind PIH1D1 and RUVBL2 (Fig. 1B). We verified that the two subcomplexes interacted in pull-down assays (fig. S1A). For cryo-EM experiments, we reconstituted a truncated R2TP complex (R2TP-ΔNT) by mixing both subcomplexes, followed by dialysis in an adenosine diphosphate (ADP)–containing buffer (Fig. 1A). The complex was vitrified and observed by cryo-EM, and all RUVBL1-RUVBL2–containing complexes were selected (fig. S1B). Typical two-dimensional (2D) class averages of R2TP-ΔNT revealed a single-ring RUVBL1-RUVBL2 structure with RPAP3-RBD domains, decorating the ATPase face of the ring as in the full R2TP complex (*17*) but without the poorly resolved conformationally heterogeneous density observed for the N-terminal regions of RPAP3 (Fig. 1C). High-resolution structural details are evident in these class averages, and our dataset comprised many orientations corresponding to tilted top and tilted side views of R2TP-ΔNT (Fig. 1D).

After 3D classification with a mask enclosing the whole molecule, most R2TP-ΔNT images could be grouped into one class containing three copies of the C-terminal RPAP3-RBD domain decorating the ATPase face of one RUVBL1-RUVBL2 hexamer and a density located at the DII face of the ring. Since RPAP3_400–665_ does not contain other globular domains besides the RBD (*17*), we assigned the density at the DII face to PIH1D1 (fig. S1C).

### Asymmetric stoichiometry of the human R2TP complex

**Fig. 2:**
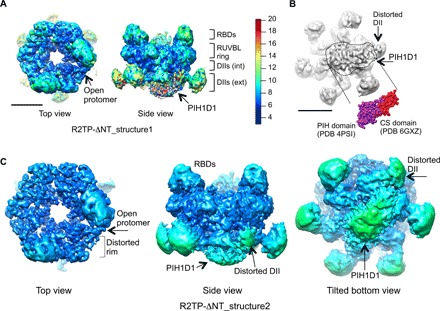
Overall architecture of R2TP-ΔNT. (**A**) Consensus 3D structure of R2TP-ΔNT at 4.0 Å resolution (R2TP-ΔNT_structure1) colored according to local resolution (*40*). The structure shows an opening in one end, as well as the location of PIH1D1 at the DII face of the ring. Color code scale is shown. (**B**) Classification of the particles based on the variability of PIH1D1 into three subgroups. The most abundant group shows clear density for PIH1D1 and the displaced DII domain, and we selected this for further refinement. A view of the DII face of the ring reveals density for PIH1D1, which agrees in shape and size with crystal structures of the PIH and CS domains, colored purple and red, respectively. Scale bar, 5 nm. (**C**) Structure of R2TP-ΔNT_structure2 obtained after refinement of the subgroup shown in (B). We estimated local resolutions in RELION (*33*), and we filtered and colored the map accordingly, using the same color code used in (A). PIH1D1, the DII domain interacting with PIH1D1, and the distorted rim of the ring are indicated. The top view is shown with a slightly higher threshold than the side and tilted views, to highlight the opening of one protomer and the distorted rim.

We refined all images to a consensus structure at an average resolution of 4.0 Å (R2TP-ΔNT_structure1) (Fig. 2A). We identified RUVBL1 and RUVBL2 subunits and the RBD domains based on previous knowledge (*17*) and after model building (see below). Higher resolution was found at the core of the RUVBL ring, whereas PIH1D1 and the external region of the DII domains are not well defined, suggesting significant flexibility and/or heterogeneity.

From this consensus refinement, we classified the dataset based on the variability of those regions where conformational changes or heterogeneity had been observed, by the use of a mask (Fig. 2B and fig. S1D). Three subgroups revealed heterogeneity in the PIH1D1 density, with the most abundant class (75.5% of the images) showing an improved density for PIH1D1 (Fig. 2B and fig. S1D). The CS domain of PIH1D1, which is sufficient to bind RPAP3 (*17*), is smaller than the N-terminal PIH domain of PIH1D1, and the resolution was sufficient to discriminate both domains and allow fitting of their crystal structures (Fig. 2B) (*7*, *8*, *11*). The density shows the presence of a single PIH1D1 molecule per RUVBL ring occupying most of the central cavity at the DII face of the ring, although the precise orientation of these small domains could not be determined at this resolution.

### Motions in PIH1D1 induce changes in the RUVBL1-RUVBL2 ring

We refined the images to 4.0 Å resolution (R2TP-ΔNT_structure2) (Fig. 2C and fig. S1E). The DII domains were visible, with the smaller region of the density ascribed to PIH1D1, possibly comprising the C-terminal CS domain in contact with a DII domain of one RUVBL subunit. This was identified as the DII domain of a RUVBL2 subunit by the presence of a bound RPAP3-RBD on the opposite face of the ring (*12*, *13*, *17*).

Interaction with PIH1D1 induces a large change in the conformation and position of this RUVBL2 DII domain, which becomes less well defined than the five other DII domains in the ring, which do not contact the PIH1D1 (Fig. 2C). These changes correlate with a distortion of the ring symmetry in the vicinity of the affected RUVBL2 subunit and the opening of the ATPase ring (Fig. 2C). These conformational changes appear to require concerted interaction of PIH1D1 and the RPAP3-RBD domain, since RBD binding alone did not alter the structure of the other RUVBL2 subunits in the ring.

**Fig. 3:**
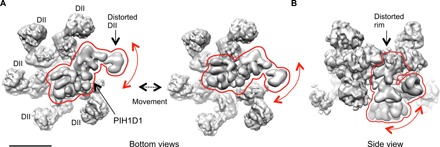
Motions of PIH1D1. (**A**) After principal components analysis, we generated 10 maps, each representing the median orientation of the particles within each specific subset. A bottom view of two of these maps is shown, highlighting the movement of PIH1D1 within the structure. The mobile element, comprising PIH1D1 and the DII domain, is enclosed within a red line. (**B**) Side view of one of the maps generated after multibody refinement. Observed motions are proximal to regions of the RUVBL1-RUVBL2 ring, suggesting that conformational changes in the DII domain could be coupled to changes in the ring. A red line is used to highlight the proximity between the mobile elements and the RUVBL1-RUVBL2 ring.

To address the flexibility of PIH1D1 and the interacting DII domain, we used a multibody refinement strategy implemented in RELION 3 (Fig. 3) (*33*). We defined three regions in R2TP-ΔNT_structure1: (i) the RUVBL1-RUVBL2 ring including the RBD domains and the internal part of the DII domains, (ii) the external segments of all DII domains except the one attached to PIH1D1, and (iii) the unit formed by PIH1D1 and the external part of the DII domain that it contacts. Multibody refinement did not improve the resolution of each of these segments compared to R2TP-ΔNT_structure2, but the analysis did reveal large relative motions of PIH1D1 and the interacting DII domain (Fig. 3A). After principal components analysis, we divided the particles into 10 subsets for each eigenvector to generate 10 maps, each representing the median orientation of the particles within each specific subset. Motions along eigenvector 2 revealed a wide range of relative orientations of PIH1D1 and the associated DII component with respect to the rest of the RUVBL1-RUVBL2 ring (Fig. 3A and movie S1). This analysis also suggested that the observed movements were coupled to the conformation of the more proximal regions of the RUVBL1-RUVBL2 ring (Fig. 3B).

### PIH1D1 induces the opening and nucleotide loss in RUVBL2

To reach the highest resolution possible, we removed the flexibility of the DII domains and PIH1D1, as well as the RBDs not involved in the open protomer, from our analysis. For this, we subtracted the density of the RUVBL1-RUVBL2 ring and the more rigid internal segments of the DII domains from each single particle, and we refined the subtracted images with a mask including only the RBD in the split protomer. This masked structure refined to an average resolution of 3.7 Å, with improved definition in the core of the RUVBL1-RUVBL2 ring (fig. S2A). In the refined maps, clear density corresponding to ADP was evident in the nucleotide-binding sites of all subunits, with the exception of the RUVBL2 subunit contacting PIHID1. This subunit showed a fainter density for the nucleotide, only detected at lower thresholds, suggesting partial occupancy.

**Fig. 4:**
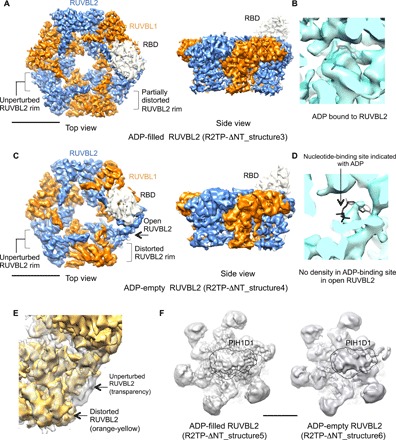
ADP-filled and ADP-empty conformations of the open RUVBL2 subunit. (**A**) Top and side view of the 3D structure of the ADP-filled open conformation of the RUVBL1-RUVBL2 ring bound to one RBD domain (R2TP-ΔNT_structure3). Color code: orange, RUVBL1; blue, RUVBL2; and white, RBD. Scale bar, 5 nm. (**B**) Close-up of the nucleotide-binding site of the open RUVBL2 subunit in R2TP-ΔNT_structure3, showing density for ADP, represented in black. (**C**) Top view and side view of the 3D structure of the ADP-empty open conformation of the RUVBL1-RUVBL2 ring bound to one RBD domain (R2TP-ΔNT_structure4). Color code: orange, RUVBL1; blue, RUVBL2; and white, RBD. Scale bar, 5 nm. (**D**) Close-up of the nucleotide-binding site of the open RUVBL2 subunit in R2TP-ΔNT_structure4, showing the absence of density in the nucleotide-binding site. ADP in the putative location of the nucleotide-binding site is represented in black to highlight the absence of density. (**E**) Close-up comparison between the rim of an unperturbed (transparent density) and a distorted RUVBL2 subunit (orange-yellow) from R2TP-ΔNT_structure5 represented at identical threshold. (**F**) Views of the DII face of the ring of the R2TP-ΔNT_structure5 and R2TP-ΔNT_structure6 structures, obtained from the particles classified as ADP-filled and ADP-empty but without subtracting the PIH1D1 region. The location of PIH1D1 in the structure is indicated within a dashed black line. Scale bar, 5 nm.

To specifically explore the heterogeneity of the asymmetric “open” conformation of the RUVBL1-RUVBL2 ring, we reclassified the images using a mask focused on the open RUVBL2 subunit. 3D classification in three subgroups (71.8, 11.8, and 16.4% of the particles in each group) revealed two major conformations, which we then refined independently. The larger group (R2TP-ΔNT_structure3, 71.8% of the particles, 3.7 Å resolution) displayed an opening and distortions of the rim of RUVBL2, which were less pronounced than in the structure obtained from all particles (Fig. 4A and fig. S2B). All subunits in the ring, including the open conformation, showed good density for ADP, indicating that despite the conformational changes, the nucleotide remains bound (Fig. 4B). We prepared samples in a buffer containing ADP in an attempt to reduce heterogeneity and increase stability during protein preparation. Thus, it is possible that the ADP bound to the open protomer might be the result of nucleotide exchange with the ADP in the buffer.

We merged the other two subgroups resulting from the 3D classification because they displayed similar conformation, and we refined them together (R2TP-ΔNT_structure4, 28.1% of the particles, 4.63 Å resolution) (Fig. 4C and fig. S2C). Whereas the overall structure of the ring was similar to R2TP-ΔNT_structure3, a much-pronounced distortion of the open RUVBL2 subunit was evident, accompanied by complete disappearance of regions corresponding to the N-terminal segment of RUVBL2 and ADP from the nucleotide-binding site (Fig. 4, D and E). The rest of the subunits in the ring showed clear density for ADP.

To test whether the ADP-filled and ADP-empty structures might correlate with a particular conformation of PIH1D1, and/or whether the ADP-filled particles might correspond to molecules that had lost PIH1D1, we re-extracted the particles for each of the two subgroups without subtraction of the PIH1D1 region, which we reprocessed (R2TP-ΔNT_structure5 and R2TP-ΔNT_structure6) (Fig. 4F). This analysis showed the presence of a similar PIH1D1 conformation, at the observed resolution, in both structures. Thus, it appears that we have captured two conformations of a PIH1D1-bound complex, both inducing changes in the RUVBL2 subunit where PIH1D1 binds, but differing in the presence or absence of ADP bound to the open RUVBL2 subunit.

### Mechanism for remodeling of the nucleotide-binding site

We built an atomic model for the ADP-filled and ADP-empty conformation of the RUVBL1-RUVBL2 ring, exploiting the presence of high-resolution features in the maps (fig. S3A). For model building, we used the information provided by the crystal structures of RUVBL1-RUVBL2, the model derived from the cryo-EM structure of the RUVBL1-RUVBL2 ring bound to the RBD domain (*17*), and the crystal structure of RUVBL2 (*26*). The resolution in the open ADP-empty RUVBL2 subunit is lower than that in the rest of the complex, and we thus only considered the secondary-structure elements for model building (see Materials and Methods for details).

**Fig. 5:**
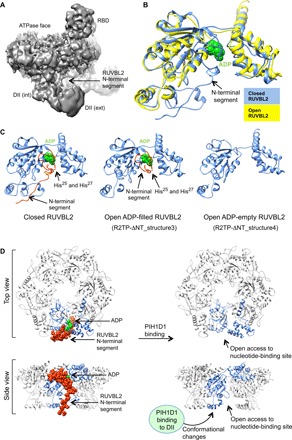
Mechanism of RUVBL1-RUVBL2 remodeling and nucleotide exchange. (**A**) A view of the map showing density for the N-terminal loop in RUVBL2 contacting the DII domain of the same protomer. (**B**) The structures of RUVBL2 closed (blue) and ADP-empty conformation (yellow) are superimposed. For alignment of the two structures, we used the neighboring RUVBL1 subunit as template. Thus, the changes observed represent movements in the context of the RUVBL ring. ADP is shown in green. (**C**) Structures of the RUVBL2 subunit in closed, open ADP-filled, and open ADP-empty conformation. ADP is shown in green. The N-terminal segment (residues 8 to 49 in the closed structure and residues 23 to 49 in the open ADP-filled structure) is colored red, indicating the position of His^25^ and His^27^. (**D**) Side view and top view of the closed and open structure of the RUVBL1-RUVBL2 hexamer, shown in ribbons, and highlighting one RUVBL2 subunit in blue. The RUVBL2 N-terminal segment shown as spheres and in red blocks the access to the nucleotide-binding site, and this obstruction is removed after PIH1D1 binds the DII domain and induces conformational changes.

During model building, we found that the distorted rim of RUVBL2 corresponded to an N-terminal segment of RUVBL2 (residues 1 to 49), which was completely lost in the ADP-empty conformation. To help during the modeling of this N-terminal segment in the “closed” RUVBL2 subunits, we applied a similar image processing strategy to that previously applied for the RBD domain (*17*). We rotated the RUVBL1-RUVBL2 ring of all particles so that all RUVBL2 subunits were placed in the same position, which were then classified and refined with a mask. The resulting map is the average of a larger number of RUVBL2 subunits in a similar conformation, thus improving structural details. During this classification, a subgroup comprising the open RUVBL2 subunit was discarded since the resolution was similar to that of the global structure. The structure of RUVBL2 obtained improved the density of the N-terminal segment in the closed RUVBL2, which approaches and likely contacts the internal segments of the DII domain of the same subunit (Fig. 5A).

The models obtained explain the molecular mechanism by which PIH1D1 binding to the DII domain of RUVBL2 is coupled to the conformational changes of the nucleotide-binding site (Fig. 5, B and C). Comparison of the conformations of closed and open RUVBL2 subunits from the ADP-empty structure of R2TP-ΔNT, after alignment of their neighboring RUVBL1 subunits, reveals the motions in the context of the RUVBL1-RUVBL2 ring, and several major areas of change became apparent (Fig. 5, B and C, and movie S2).

First, changes in the position of several α helices suggest that the torsions of the DII-external domain accompanying its interaction with PIH1D1 are transmitted to the internal region of the DII domain, and this relays the changes to elements essential for nucleotide binding and nucleotide exchange (Fig. 5B and movie S2). Second, in the closed RUVBL2 protomers, an N-terminal segment (residues 1 to 49) that interacts with bound ADP via two histidine residues (His^25^ and His^27^) also contacts the DII domain (Fig. 5C). These two histidine residues are defined in the cryo-EM density, pointing toward the ribose and the base of the ADP (fig. S3B). Residues 28 to 49 of the N-terminal segment are also visible in the cryo-EM map (fig. S3C). In the open ADP-empty RUVBL2 subunit, the density for this N-terminal segment disappears completely, and the contacts with the DII domain and the nucleotide are lost (fig. S3D). The open ADP-filled RUVBL2 subunit shows an intermediate level of order such that density is still evident for the two histidine residues that contact the ADP but absent for the upstream segment that interacts with the DII domain in the closed RUVBL2 protomers (Fig. 5C). Collectively, these results show that structural changes in the DII domain that accompany binding of PIH1D1 control access to the nucleotide-binding site (Fig. 5D).

## DISCUSSION

The heterohexameric RUVBL1-RUVBL2 ring is the most highly conserved and the most consistently essential component of all R2TP and all R2TP-like complexes so far described (*12*, *13*). Despite this, the biological role and regulatory mechanism of its inherent ATPase activity are poorly understood. Here, we show that the other components of the R2TP complex play a direct role in regulating the conformation of the nucleotide-binding sites in the RUVBL1-RUVBL2 ring.

**Fig. 6:**
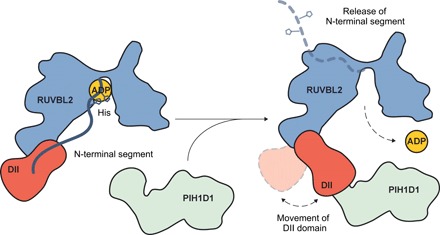
Model for remodeling of RUVBL1-RUVBL2 by PIH1D1. Cartoon for the mechanistic model of how PIH1D1 regulates the accessibility to the nucleotide-binding site of RUVBL1-RUVBL2.

Crystal and cryo-EM structures of yeast and mammalian RUVBL1-RUVBL2 complexes show that the nucleotides bound to both types of subunit are effectively buried within the heterohexameric ring. Hexamerization blocks the nucleotide-binding pocket, and ADP and/or ATP are trapped in the complex during purification (*22*, *32*). An active ATPase cycle therefore requires passage through an open conformation to allow exchange of bound ADP for ATP. Our results define a mechanism whereby binding of PIH1D1, the client recruitment component of the R2TP complex, induces a conformational change in the external region of the DII domain of a RUVBL2 subunit. This is transmitted to the internal DII region and coupled to changes in the nucleotide-binding site, resulting in the opening of the ring and loss of the nucleotide (Fig. 6).

In this mechanism, the N-terminal segment of RUVBL2 functions as a gatekeeper for the nucleotide-binding site. In the closed state, this segment is ordered via interaction with the internal part of the DII domain of the same subunit and blocks access to the nucleotide-binding site. A similar interaction between the N terminus of RUVBL2 and the DII domain has also been observed in the crystal structure of an Rvb1/Rvb2 complex from *Chaetomium thermophilum* (*20*). In the closed conformation, two histidine residues (His^25^ and His^27^) interact with the nucleotide. Several crystal structures of RUVBL1/Rvb1-RUVBL2/Rvb2 show that these two residues participate in nucleotide binding but not in hydrolysis (*15*, *16*, *20*, *22*, *34*). Binding of PIH1D1 to the external part of the DII domain induces large motions in the RUVBL2 subunit, resulting in an order-disorder transition in the N-terminal segment, which opens the “gate” to the nucleotide-binding site to facilitate nucleotide exchange. Although not previously described in RUVBL1-RUVBL2, asymmetric opening of AAA ATPase rings has been observed in other systems such as the VAT unfoldase from *Thermoplasma acidophilum* (*35*) and the complex of p31^comet^-C-MAD2 with TRIP13 (*36*).

Our data show that PIH1D1 promotes and stabilizes the transient open state of the nucleotide-binding domain. RUVBL1-RUVBL2 binds to nucleotides for its known cellular functions (*16*, *30*, *31*, *34*), but the exact role of nucleotide-binding exchange and/or hydrolysis in R2TP is unknown. The conformational changes in RUVBL1-RUVBL2 induced by PIH1D1 could help in the coupling of client recruitment to the regulation of nucleotide exchange. It cannot be discarded that it could be a preformed PIH1D1-client complex that interacts and regulates RUVBL1-RUVBL2. The open conformation induced by PIH1D1 would facilitate the release of bound nucleotides and the access or exchange of nucleotides. It is likely that subsequent binding of ATP to the PIH1D1-promoted open nucleotide-binding site could trigger ring closure. Whether this is accompanied by direct reversal of the conformational change in the DII domain elicited by binding of PIH1D1, which could then affect the conformation of the clients, or by full disengagement of PIH1D1 from the complex is currently unknown.

Besides interacting with RPAP3 and the RUVBL2 through its CS domain, PIH1D1 also interacts with factors such as TELO2 and MRE11 through its PIH domain (*7*, *8*, *27*). The presence of additional components bound to PIH1D1 is likely to affect its function as a nucleotide exchange factor and may provide a route by which substrates and substrate adaptors can modulate the RUVBL ATPase activity within R2TP complexes. However, the requirement for a nucleotide exchange factor for RUVBL1-RUVBL2 is not absolute, and R2T complexes containing RPAP3 isoform 2, which lacks the PIH1D1-binding segment, have been identified in cell extracts (*13*), although their biological function has not been defined. R2TP-like complexes containing PIH1D1-related proteins (PIH1D2, PIH1D3, and DNAAF2) have also been identified (*13*). Our structure suggests that it is the CS domain of PIH1D1 that binds RUVBL2. This domain is highly conserved between the PIH1D1-related proteins, and thus, it is possible that these could also induce conformational changes in RUVBL1-RUVBL2.

We and others previously determined the cryo-EM structure of the yeast R2TP complex, in which a single copy of a Tah1p-Pih1p heterodimer was bound to the DII domain face of the Rvb1-Rvb2 ring (*24*, *25*). Previous cryo-EM analysis of the human R2TP complex, in which Tah1p is replaced by the much larger TPR-domain protein RPAP3, was unable to define the number of PIH1D1 subunits bound per complex due to interference of the flexible N-terminal parts of RPAP3 in image analysis. Human R2TP contains up to three RPAP3 molecules, one for each RUVBL2 in the complex (*17*). Since each RPAP3 can potentially interact with one PIH1D1, whether the human R2TP complex comprises one copy or several copies of PIH1D1 remains undefined. Using a truncated RPAP3 that retains the ability to bind RUVBL1-RUVBL2 and PIH1D1, we now determine unambiguously that as in yeast, only a single copy of PIH1D1 is bound to the DII face of the human RUVBL1-RUVBL2 ring.

Unlike the yeast R2TP system, where interaction of Pih1p with the Rvb1-Rvb2 proteins mediates recruitment of Tah1p (*24*), stable retention of PIH1D1 within the human R2TP complex is dependent on its constitutive interaction with a central segment of RPAP3 (*11*, *17*). Recruitment of RPAP3 itself depends on the interaction of its C-terminal RBD domain with the ATPase face of the RUVBL1-RUVBL2 ring (*17*). While our structural data (see above) show that only a single asymmetrically bound PIH1D1 molecule can be accommodated on the DII face of the ring, there is no such steric restriction on the opposite face of the ring, where the RPAP3-RBD binds to the ATPase domain of RUVBL2. We commonly observe well-ordered density for up to three RPAP3-RBD domains in human R2TP complexes, suggesting that three RPAP3 molecules are present in the complex. Given their tight physical association, it is reasonable to assume that all three RPAP3 molecules carry a PIH1D1 molecule, but only one at a time is able to take up the conformation in which they wrap around the edge of the ring with their associated PIH1D1 binding to a RUVBL2 DII domain. How these multiple bound copies of RPAP3-PIH1D1 function in the presence of collaborating proteins such as HSP90 and/or the TTT complex and how this contributes to the assembly of the various multiprotein complexes that depend on the extended HSP90-R2TP chaperone system remain to be determined.

Our results show that protein interactions in the DII face of the ring relay “information” to the ATPase domain to regulate nucleotide access, and this information could potentially flow in both directions. While this is an important advance in understanding RUVBL1-RUVBL2 regulation within the R2TP co-chaperone, conformational changes induced in the DII domains by the interaction of client proteins can propagate to the nucleotide-binding sites in other RUVBL1-RUVBL2–containing complexes. An insertion of the INO80 chromatin remodeling ATPase subunit of the yeast INO80 complex interacts with Rvb1-Rvb2 and induces conformational changes in the DII and AAA domains (*15*). Similarly, structures of human INO80 also reveal conformational changes in the DII domains and the loss of a perfect symmetry in the hexameric ring (*34*, *37*). Collectively, these structures support the idea that client proteins interact and regulate the conformation of the DII domains and that these changes affect the AAA ring.

The structures of yeast and human INO80 (*15*, *34*, *37*) and yeast SWR1 (*16*) show that the nucleotide remains trapped in all subunits. In these structures, the N-terminal regions of RUVBL1(Rvb1)/RUVBL2(Rvb2) are ordered, and the N-terminal histidine residues interact with the nucleotide, as in the closed conformation of RUVBL2 we describe. These structures show that clients induce changes in the DII domain and the AAA region, but they do not explain how a small insertion of the IN080 chromatin remodeling ATPase subunit of yeast INO80 stimulates the ATPase activity of Rvb1-Rvb2 (*30*). It is possible that after all the correct set of interactions has taken place, as in the cryo-EM structure, the INO80 complex assembles a stable form with ADP bound. Also, the specific regulatory function of the N-terminal segment of RUVBL1-RUVBL2 that we find for R2TP might not operate for INO80 complexes. Despite the specificities of each complex, the coupling of protein-induced motions of the DII domains with the conformation of the nucleotide-binding site appears as a general mechanism shared by many RUVBL1-RUVBL2–containing complexes.

## MATERIALS AND METHODS

### Cloning

We cloned human *RPAP3*_*400–66*5_, *RUVBL1*, *RUVBL2*, and *PIH1D1* as indicated in Martino *et al*. (*17*). RPAP3 and PIH1D1 were cloned containing an N-terminal GST and 6×His-tag, respectively. We purchased *RPAP3* from GenScript, and we cloned N-terminal truncations into a pGex6p-modified plasmid named p3E (University of Sussex, UK) using infusion cloning (Clontech Laboratories Inc.) (table S1).

We produced RUVBL1-RUVBL2 complexes using RUVBL1 cloned together with RUVBL2. RUVBL1 contained an N-terminal 6×His-tag whereas the RUVBL2 subunit was untagged. There is no evidence that the His-tag at the N terminus of RUVBL1 affects the structure or functions of RUVBL1-RUVBL2 (*17*, *21*).

### Protein expression and purification

For cryo-EM, we produced the human RUVBL1-RUVBL2 protein complex, as described previously (*17*, *21*). Affinity purification using a nickel column (His-tag purification) was followed by Sephacryl S300 (GE Healthcare) size-exclusion chromatography in a buffer containing 50 mM tris, 300 mM NaCl, and 1 mM dithiothreitol. The purified RUVBL1-RUVBL2 complexes used for cryo-EM contain a His-tag at the N terminus of RUVBL1, but the RUVBL2 subunit, with which PIH1D1 interacts, does not have a tag. For pull-down experiments, we cloned a RUVBL1-RUVBL2 complex without tag in RUVBL1 and a C-terminal strep-tag in RUVBL2 and purified it by affinity purification, followed by gel filtration.

We purified the RPAP3_400–665_-PIH1D1 complex, as described previously (*17*). Briefly, we used glutathione *S*-transferase (GST)–tag affinity chromatography to bind GST-RPAP3_400–665_, and we eluted the protein using 50 mM glutathione. We incubated the elution with PreScission protease (3C protease) at 4°C overnight to remove the tag, followed by a subsequent gel filtration chromatography using an S200 26/60 column. We reconstituted R2TP-ΔNT by mixing RUVBL1-RUVBL2 and RPAP3_400–665_-PIH1D1 subcomplexes in a 1:4 molar ratio, followed by dialysis in 25 mM Hepes (pH 7.8), 130 mM NaCl, and 10 mM 2-mercaptoethanol–containing buffer containing 0.5 mM ADP (pH 7.0) at 4°C for 4 hours.

### Pull-down experiments

We mixed 42 μM RPAP3_400–665_-PIH1D1 subcomplex containing an N-terminal His-tag in PIH1D1 and 25 μM RUVBL1-RUVBL2 containing a C-terminal strep-tag in RUVBL2 with 100 μl of StrepTactin Sepharose (General Electric) equilibrated in 25 mM Hepes (pH 7.8), 130 mM NaCl, and 0.5 mM tris(2-carboxyethyl)phosphine (TCEP). We incubated this reaction mixture for 2 hours at 4°C, followed by two washes with buffer and an elution using the same buffer but complemented with 2.5 mM desthiobiotin.

### Cryo-EM of human R2TP-ΔNT

Crystal structures have shown that nucleotides bind RUVBL1-RUVBL2 (and the homologous proteins Rvb1-Rvb2) without the need of magnesium and that nucleotides can be trapped within the structure of RUVBL1-RUVBL2 during purification (*22*, *32*). After dialysis, we incubated R2TP-ΔNT with 0.5 mM ADP (pH 7.0) for 2 hours to ensure that the presence of ADP is not limiting for the available nucleotide-binding sites in the complex. Then, we applied 3.7 μl of the complex to Quantifoil 300 mesh R1.2/1.3 grids after glow discharge, and the complex was flash frozen in liquid ethane using FEI Vitrobot MAG IV (Thermo Fisher Scientific). We collected 4077 movies using a GATAN K2 Summit detector, counting mode, and a slit width of 20 eV on a GIF Quantum energy filter in a Titan Krios at the Astbury Centre for Structural Molecular Biology (Leeds, UK). Microscope calibrations and automatic data acquisition were performed with EPU software (Thermo Fisher Scientific). The acquisition conditions were as follows: nominal magnification of ×130,000, 70-mm C2 aperture, spot size of 7, illuminated area of 1.35 μm, zero-loss imaging with 20-eV slit, physical pixel size of 1.07 Å, a total dose of 47.5 e^−^/Å^2^, 4.75 e^−^/Å^2^/s, a total exposure time of 10 s, and 40 fractions. Autofocus was performed every 10 μm using an objective defocus range between −1.5 and −2.5 μm (table S2).

### Image processing

We used MotionCorr2 (*38*) for whole-frame motion correction, GCTF (*39*) for estimation of the contrast transfer function parameters, and Gautomatch [Jack (Kai) Zhang at www.mrc-lmb.cam.ac.uk/kzhang/] and CTF refinement for beam tilt estimation for particle selection. Particles were then processed using RELION 3.0 (*33*). We first classified a total dataset of 408,781 particles to discard images that did not correspond to molecule images (fig. S1C). Then, we processed the particles in rounds of 3D classification and refinement following standard methodologies, as well as CTF refinement with beam tilt estimation. After a consensus refinement, image processing followed two separate strategies (fig. S1C). On the one hand, we classified the particles based on the heterogeneity of the PIH1D1 region, and we refined the selected subgroups (fig. S1, C and D). We processed these particles using a multibody refinement strategy, as defined in RELION 3 (*33*). We selected user-defined region boundaries as indicated in the main text, and we analyzed and interpreted the results as described by Nakane *et al.* (*33*). Details for the resolution and number of particles for each structure are provided in table S3.

On the other hand, we subtracted the RUVBL ring including the RBD in the open protomer and the internal DII domains, and we refined these particles independently to an estimated resolution of 3.7 Å [using a cutoff of FSC (Fourier shell correlation) = 0.143 and gold standard FSC] (fig. S2A). The structure was split into an ADP-empty and an ADP-filled conformation, after a 3D classification focused in the conformation of the open RUVBL2 subunit.

We obtained an improved density for the N-terminal loop of RUVBL2 by applying the processing strategy used in the RBD domain in previous work for the R2TP complex (*17*). After rotation of the RUVBL ring in every particle by 120° and 240°, all DII domains converge to the same location, thus increasing the number of images to process and average. After 3D classification, we selected the best images and we refined them, showing improved density for the N-terminal end of RUVBL2. Similar densities can be traced in the closed RUVBL2 subunits in the other structures solved, but their observation was more dependent in the threshold level used and degree of sharpening. We classified some particles as DII domains corresponded to the open RUVBL2 subunit, but we did not refine these further, as the number of images was the same than in the whole structure, and the resolution did not improve. Sharpening was performed using automatic procedures in RELION 2. We used ResMap (*40*) and RELION (*33*) to estimate local resolutions. For interpretation of PIH1D1, we used the structure of the CS domain of human PIH1D1 from the RPAP3(TPR2)-PIH1D1(CS) complex [Protein Data Bank (PDB) 6GXZ] (*11*) and the PIH N-terminal domain from mouse (PDB 4CKT) (*7*).

### Model building

We built a model using the following information: the structure of the RBD domain bound to RUVBL1-RUVBL2 (PDB 6FO1), the crystal structure of RUVBL1 (PDB 2C9O), and the crystal structure of RUVBL2 (PDB 6H7X). For the model of the open ring, we fitted the structure of the RUVBL1-RUVBL2 in the R2TP complex (PDB 6FO1) into the cryo-EM map, and model building was performed using Coot (*41*) and Phenix (*42*). We used rigid fitting of the RUVBL1-RUVBL2 ring from PDB model 6FO1 as input for real space refinement in R2TP-ΔNT_structure3 (ADP-filled) in Phenix, using a minimization for 10 macro cycles as the default protocol for all the real space refinements performed, and the resolution limit for R2TP-ΔNT_structure3 (ADP-filled) is 4 Å. The output model was suitable for manual refinement in Coot for all the six chains present in the model using the same R2TP-ΔNT_structure3 map. Another round of Phenix real space refinement led to a correct model in terms of statistics (table S4) and overall good fitting in R2TP-ΔNT_structure3 (ADP-filled). We modeled the N terminus of RUVBL2 in its closed conformation from residues 8 to 49 and from residues 23 to 49 in the ADP-filled open conformation, with the help of the cryo-EM map of the averaged DII domains (Fig. 5A), as well as the unsharpened version of R2TP-ΔNT_structure3 (ADP-filled).

The resolution of the R2TP-ΔNT_structure4 (ADP-empty) structure was lower than that of the ADP-filled structure. To build the R2TP-ΔNT_structure4 (ADP-empty) model, we used the R2TP-ΔNT_structure3 (ADP-filled) model as a starting point for a new real space refinement in Phenix against the R2TP-ΔNT_structure4 (ADP-empty) map and set the resolution limit to 6 Å, excluding the ADP molecule and the N-terminal loop for the RUVBL2 (which is not observed in this structure) in the model. The statistics of this model are also shown in table S4.

## Supplementary Material

http://advances.sciencemag.org/cgi/content/full/5/5/eaaw1616/DC1

Download PDF

Movie S1

Movie S2

## SUPPLEMENTARY MATERIALS

Supplementary material for this article is available at http://advances.sciencemag.org/cgi/content/full/5/5/eaaw1616/DC1 Table S1. Primers used for cloning in this work. Table S2. Conditions used for cryo-EM data collection for all the cryo-EM maps in this work. Table S3. Cryo-EM data refinement and validation statistics for all the cryo-EM maps in this work. Table S4. Validation statistics for the atomic models for R2TP-ΔNT_structure3 and R2TP-ΔNT_structure4. Fig. S1. Cryo-EM of truncated human R2TP (R2TP-ΔNT). Fig. S2. Resolution estimation for cryo-EM structures obtained from the subtracted particles. Fig. S3. High-resolution details in the cryo-EM maps. Movie S1. Several views of the multibody refinement strategy, showing the motions of the PIH1D1 region bound to a DII domain of RUVBL2, with respect to the rest of the molecule. Movie S2. Conformational changes in the RUVBL2 subunit, from a closed conformation to an open ADP-filled conformation to an open ADP-empty conformation.

## References

[1] Machado-Pinilla R., Liger D., Leulliot N., Meier U. T., Mechanism of the AAA+ ATPases pontin and reptin in the biogenesis of H/ACA RNPs. RNA 18, 1833–1845 (2012). 22923768 10.1261/rna.034942.112 PMC3446707

[2] Horejsi Z., Takai H., Adelman C. A., Collis S. J., Flynn H., Maslen S., Skehel J. M., de Lange T., Boulton S. J., CK2 phospho-dependent binding of R2TP complex to TEL2 is essential for mTOR and SMG1 stability. Mol. Cell 39, 839–850 (2010). 20864032 10.1016/j.molcel.2010.08.037

[3] Cloutier P., Poitras C., Durand M., Hekmat O., Fiola-Masson É., Bouchard A., Faubert D., Chabot B., Coulombe B., R2TP/Prefoldin-like component RUVBL1/RUVBL2 directly interacts with ZNHIT2 to regulate assembly of U5 small nuclear ribonucleoprotein. Nat. Commun. 8, 15615 (2017). 28561026 10.1038/ncomms15615 PMC5460035

[4] Kim S. G., Hoffman G. R., Poulogiannis G., Buel G. R., Jang Y. J., Lee K. W., Kim B. Y., Erikson R. L., Cantley L. C., Choo A. Y., Blenis J., Metabolic stress controls mTORC1 lysosomal localization and dimerization by regulating the TTT-RUVBL1/2 complex. Mol. Cell 49, 172–185 (2013). 23142078 10.1016/j.molcel.2012.10.003 PMC3545014

[5] Takai H., Xie Y., de Lange T., Pavletich N. P., Tel2 structure and function in the Hsp90-dependent maturation of mTOR and ATR complexes. Genes Dev. 24, 2019–2030 (2010). 20801936 10.1101/gad.1956410 PMC2939364

[6] Matias P. M., Baek S. H., Bandeiras T. M., Dutta A., Houry W. A., Llorca O., Rosenbaum J., The AAA+ proteins Pontin and Reptin enter adult age: From understanding their basic biology to the identification of selective inhibitors. Front. Mol. Biosci. 2, 17 (2015). 25988184 10.3389/fmolb.2015.00017 PMC4428354

[7] Pal M., Morgan M., Phelps S. E., Roe S. M., Parry-Morris S., Downs J. A., Polier S., Pearl L. H., Prodromou C., Structural basis for phosphorylation-dependent recruitment of Tel2 to Hsp90 by Pih1. Structure 22, 805–818 (2014). 24794838 10.1016/j.str.2014.04.001 PMC4058522

[8] Hořejší Z., Stach L., Flower T. G., Joshi D., Flynn H., Skehel J. M., O'Reilly N. J., Ogrodowicz R. W., Smerdon S. J., Boulton S. J., Phosphorylation-dependent PIH1D1 interactions define substrate specificity of the R2TP cochaperone complex. Cell Rep. 7, 19–26 (2014). 24656813 10.1016/j.celrep.2014.03.013 PMC3989777

[9] Cloutier P., Al-Khoury R., Lavallée-Adam M., Faubert D., Jiang H., Poitras C., Bouchard A., Forget D., Blanchette M., Coulombe B., High-resolution mapping of the protein interaction network for the human transcription machinery and affinity purification of RNA polymerase II-associated complexes. Methods 48, 381–386 (2009). 19450687 10.1016/j.ymeth.2009.05.005 PMC4492713

[10] Boulon S., Pradet-Balade B., Verheggen C., Molle D., Boireau S., Georgieva M., Azzag K., Robert M. C., Ahmad Y., Neel H., Lamond A. I., Bertrand E., HSP90 and its R2TP/Prefoldin-like cochaperone are involved in the cytoplasmic assembly of RNA polymerase II. Mol. Cell 39, 912–924 (2010). 20864038 10.1016/j.molcel.2010.08.023 PMC4333224

[11] Henri J., Chagot M. E., Bourguet M., Abel Y., Terral G., Maurizy C., Aigueperse C., Georgescauld F., Vandermoere F., Saint-Fort R., Behm-Ansmant I., Charpentier B., Pradet-Balade B., Verheggen C., Bertrand E., Meyer P., Cianferani S., Manival X., Quinternet M., Deep structural analysis of RPAP3 and PIH1D1, two components of the HSP90 co-chaperone R2TP complex. Structure 26, 1196–1209.e8 (2018). 30033218 10.1016/j.str.2018.06.002

[12] Coulombe B., Cloutier P., Gauthier M. S., How do our cells build their protein interactome? Nat. Commun. 9, 2955 (2018). 30054485 10.1038/s41467-018-05448-2 PMC6063932

[13] Maurizy C., Quinternet M., Abel Y., Verheggen C., Santo P. E., Bourguet M., Paiva A. C. F., Bragantini B., Chagot M.-E., Robert M.-C., Abeza C., Fabre P., Fort P., Vandermoere F., Sousa P. M. F., Rain J.-C., Charpentier B., Cianférani S., Bandeiras T. M., Pradet-Balade B., Manival X., Bertrand E., The RPAP3-Cterminal domain identifies R2TP-like quaternary chaperones. Nat. Commun. 9, 2093 (2018). 29844425 10.1038/s41467-018-04431-1 PMC5974087

[14] Houry W. A., Bertrand E., Coulombe B., The PAQosome, an R2TP-based chaperone for quaternary structure formation. Trends Biochem. Sci. 43, 4–9 (2018). 29203338 10.1016/j.tibs.2017.11.001

[15] Eustermann S., Schall K., Kostrewa D., Lakomek K., Strauss M., Moldt M., Hopfner K. P., Structural basis for ATP-dependent chromatin remodelling by the INO80 complex. Nature 556, 386–390 (2018). 29643509 10.1038/s41586-018-0029-y PMC6071913

[16] WillhoftO., GhoneimM., LinC. L., ChuaE. Y. D., WilkinsonM., ChabanY., AyalaR., McCormackE. A., OclooL., RuedaD. S., WigleyD. B., Structure and dynamics of the yeast SWR1-nucleosome complex. Science 362, eaat7716 (2018).3030991810.1126/science.aat7716

[17] Martino F., Pal M., Muñoz-Hernández H., Rodríguez C. F., Núñez-Ramírez R., Gil-Carton D., Degliesposti G., Skehel J. M., Roe S. M., Prodromou C., Pearl L. H., Llorca O., RPAP3 provides a flexible scaffold for coupling HSP90 to the human R2TP co-chaperone complex. Nat. Commun. 9, 1501 (2018). 29662061 10.1038/s41467-018-03942-1 PMC5902453

[18] Massenet S., Bertrand E., Verheggen C., Assembly and trafficking of box C/D and H/ACA snoRNPs. RNA Biol. 14, 680–692 (2017). 27715451 10.1080/15476286.2016.1243646 PMC5519232

[19] Ewens C. A., Su M., Zhao L., Nano N., Houry W. A., Southworth D. R., Architecture and nucleotide-dependent conformational changes of the Rvb1-Rvb2 AAA+ complex revealed by cryoelectron microscopy. Structure 24, 657–666 (2016). 27112599 10.1016/j.str.2016.03.018 PMC13429012

[20] Lakomek K., Stoehr G., Tosi A., Schmailzl M., Hopfner K. P., Structural basis for dodecameric assembly states and conformational plasticity of the full-length AAA+ ATPases Rvb1. Rvb2. Structure 23, 483–495 (2015). 25661652 10.1016/j.str.2014.12.015

[21] López-Perrote A., Muñoz-Hernández H., Gil D., Llorca O., Conformational transitions regulate the exposure of a DNA-binding domain in the RuvBL1-RuvBL2 complex. Nucleic Acids Res. 40, 11086–11099 (2012). 23002137 10.1093/nar/gks871 PMC3510503

[22] Gorynia S., Bandeiras T. M., Pinho F. G., McVey C. E., Vonrhein C., Round A., Svergun D. I., Donner P., Matias P. M., Carrondo M. A., Structural and functional insights into a dodecameric molecular machine—The RuvBL1/RuvBL2 complex. J. Struct. Biol. 176, 279–291 (2011). 21933716 10.1016/j.jsb.2011.09.001

[23] Cheung K. L., Huen J., Houry W. A., Ortega J., Comparison of the multiple oligomeric structures observed for the Rvb1 and Rvb2 proteins. Biochem. Cell Biol. 88, 77–88 (2010). 20130681 10.1139/o09-159 PMC2980847

[24] Rivera-Calzada A., Pal M., Muñoz-Hernández H., Luque-Ortega J. R., Gil-Carton D., Degliesposti G., Skehel J. M., Prodromou C., Pearl L. H., Llorca O., The structure of the R2TP complex defines a platform for recruiting diverse client proteins to the HSP90 molecular chaperone system. Structure 25, 1145–1152.e4 (2017). 28648606 10.1016/j.str.2017.05.016 PMC5501727

[25] Tian S., Yu G., He H., Zhao Y., Liu P., Marshall A. G., Demeler B., Stagg S. M., Li H., Pih1p-Tah1p puts a lid on hexameric AAA+ ATPases Rvb1/2p. Structure 25, 1519–1529.e4 (2017). 28919439 10.1016/j.str.2017.08.002 PMC6625358

[26] Silva S. T. N., Brito J. A., Arranz R., Sorzano C. Ó. S., Ebel C., Doutch J., Tully M. D., Carazo J.-M., Carrascosa J. L., Matias P. M., Bandeiras T. M., X-ray structure of full-length human RuvB-Like 2—Mechanistic insights into coupling between ATP binding and mechanical action. Sci. Rep. 8, 13726 (2018). 30213962 10.1038/s41598-018-31997-z PMC6137109

[27] von Morgen P., Burdova K., Flower T. G., O'Reilly N. J., Boulton S. J., Smerdon S. J., Macurek L., Horejsi Z., MRE11 stability is regulated by CK2-dependent interaction with R2TP complex. Oncogene 36, 4943–4950 (2017). 28436950 10.1038/onc.2017.99 PMC5531254

[28] Vaughan C. K., Hsp90 picks PIKKs via R2TP and Tel2. Structure 22, 799–800 (2014). 24918336 10.1016/j.str.2014.05.012 PMC4058748

[29] Takai H., Wang R. C., Takai K. K., Yang H., de Lange T., Tel2 regulates the stability of PI3K-related protein kinases. Cell 131, 1248–1259 (2007). 18160036 10.1016/j.cell.2007.10.052

[30] Zhou C. Y., Stoddard C. I., Johnston J. B., Trnka M. J., Echeverria I., Palovcak E., Sali A., Burlingame A. L., Cheng Y., Narlikar G. J., Regulation of Rvb1/Rvb2 by a domain within the INO80 chromatin remodeling complex implicates the yeast Rvbs as protein assembly chaperones. Cell Rep. 19, 2033–2044 (2017). 28591576 10.1016/j.celrep.2017.05.029 PMC5564220

[31] Izumi N., Yamashita A., Iwamatsu A., Kurata R., Nakamura H., Saari B., Hirano H., Anderson P., Ohno S., AAA+ proteins RUVBL1 and RUVBL2 coordinate PIKK activity and function in nonsense-mediated mRNA decay. Sci. Signal. 3, ra27 (2010). 20371770 10.1126/scisignal.2000468

[32] Matias P. M., Gorynia S., Donner P., Carrondo M. A., Crystal structure of the human AAA+ protein RuvBL1. J. Biol. Chem. 281, 38918–38929 (2006). 17060327 10.1074/jbc.M605625200

[33] Nakane T., Kimanius D., Lindahl E., Scheres S. H., Characterisation of molecular motions in cryo-EM single-particle data by multi-body refinement in RELION. eLife 7, e36861 (2018). 29856314 10.7554/eLife.36861 PMC6005684

[34] Ayala R., Willhoft O., Aramayo R. J., Wilkinson M., McCormack E. A., Ocloo L., Wigley D. B., Zhang X., Structure and regulation of the human INO80-nucleosome complex. Nature 556, 391–395 (2018). 29643506 10.1038/s41586-018-0021-6 PMC5937682

[35] Ripstein Z. A., Huang R., Augustyniak R., Kay L. E., Rubinstein J. L., Structure of a AAA+ unfoldase in the process of unfolding substrate. eLife 6, e25754 (2017). 28390173 10.7554/eLife.25754 PMC5423775

[36] Alfieri C., Chang L., Barford D., Mechanism for remodelling of the cell cycle checkpoint protein MAD2 by the ATPase TRIP13. Nature 559, 274–278 (2018). 29973720 10.1038/s41586-018-0281-1 PMC6057611

[37] Aramayo R. J., Willhoft O., Ayala R., Bythell-Douglas R., Wigley D. B., Zhang X., Cryo-EM structures of the human INO80 chromatin-remodeling complex. Nat. Struct. Mol. Biol. 25, 37–44 (2018). 29323271 10.1038/s41594-017-0003-7 PMC5777635

[38] Zheng S. Q., Palovcak E., Armache J.-P., Verba K. A., Cheng Y., Agard D. A., MotionCor2: Anisotropic correction of beam-induced motion for improved cryo-electron microscopy. Nat. Methods 14, 331–332 (2017). 28250466 10.1038/nmeth.4193 PMC5494038

[39] Zhang K., Gctf: Real-time CTF determination and correction. J. Struct. Biol. 193, 1–12 (2016). 26592709 10.1016/j.jsb.2015.11.003 PMC4711343

[40] Kucukelbir A., Sigworth F. J., Tagare H. D., Quantifying the local resolution of cryo-EM density maps. Nat. Methods 11, 63–65 (2014). 24213166 10.1038/nmeth.2727 PMC3903095

[41] Emsley P., Lohkamp B., Scott W. G., Cowtan K., Features and development of Coot. Acta Crystallogr. D Biol. Crystallogr. 66, 486–501 (2010). 20383002 10.1107/S0907444910007493 PMC2852313

[42] Adams P. D., Afonine P. V., Bunkoczi G., Chen V. B., Davis I. W., Echols N., Headd J. J., Hung L. W., Kapral G. J., Grosse-Kunstleve R. W., McCoy A. J., Moriarty N. W., Oeffner R., Read R. J., Richardson D. C., Richardson J. S., Terwilliger T. C., Zwart P. H., PHENIX: A comprehensive Python-based system for macromolecular structure solution. Acta Crystallogr. D Biol. Crystallogr. 66, 213–221 (2010). 20124702 10.1107/S0907444909052925 PMC2815670

