# Supplementary material for: Structural mechanism for regulation of the AAA-ATPases RUVBL1-RUVBL2 in the R2TP co-chaperone revealed by cryo-EM
Source: Sci Adv. 2019 May 1;5(5):eaaw1616. doi: 10.1126/sciadv.aaw1616 (PMC6494491; doi:10.1126/sciadv.aaw1616)
Supplement: http://advances.sciencemag.org/cgi/content/full/5/5/eaaw1616/DC1 [file supp_5_5_eaaw1616__index.html]

Science Advances | Science Advances

## Supplementary Materials

**The PDF file includes:**

- Table S1. Primers used for cloning in this work.
- Table S2. Conditions used for cryo-EM data collection for all the cryo-EM maps in this work.
- Table S3. Cryo-EM data refinement and validation statistics for all the cryo-EM maps in this work.
- Table S4. Validation statistics for the atomic models for R2TP-ΔNT\_structure3 and R2TP-ΔNT\_structure4.
- Fig. S1. Cryo-EM of truncated human R2TP (R2TP-ΔNT).
- Fig. S2. Resolution estimation for cryo-EM structures obtained from the subtracted particles.
- Fig. S3. High-resolution details in the cryo-EM maps.
- Legends for movies S1 and S2

Download PDF

**Other Supplementary Material for this manuscript includes the following:**

- Movie S1 (.mov format). Several views of the multibody refinement strategy, showing the motions of the PIH1D1 region bound to a DII domain of RUVBL2, with respect to the rest of the molecule.
- Movie S2 (.mp4 format). Conformational changes in the RUVBL2 subunit, from a closed conformation to an open ADP-filled conformation to an open ADP-empty conformation.

**Files in this Data Supplement:**

- Adobe PDF - aaw1616\_SM.pdf
