# Supplementary material for: Structural mechanism for regulation of the AAA-ATPases RUVBL1-RUVBL2 in the R2TP co-chaperone revealed by cryo-EM
Source: Sci Adv. 2019 May 1;5(5):eaaw1616. doi: 10.1126/sciadv.aaw1616 (PMC6494491; doi:10.1126/sciadv.aaw1616)
Supplement: Download PDF [file aaw1616_SM.pdf]

Science Advances
AAAS

advances.sciencemag.org/cgi/content/full/5/5/eaaw1616/DC1

Supplementary Materials for

Structural mechanism for regulation of the AAA-ATPases RUVBL1-RUVBL2 in the R2TP co-chaperone revealed by cryo-EM

Hugo Muñoz-Hernández, Mohinder Pal, Carlos F. Rodríguez, Rafael Fernandez-Leiro, Chrisostomos Prodromou, Laurence H. Pearl, Oscar Llorca*

*Corresponding author. Email: ollorca@cnio.es

Published 1 May 2019, *Sci. Adv.* **5**, eaaw1616 (2019)
DOI: 10.1126/sciadv.aaw1616

The PDF file includes:

Table S1. Primers used for cloning in this work.
Table S2. Conditions used for cryo-EM data collection for all the cryo-EM maps in this work.
Table S3. Cryo-EM data refinement and validation statistics for all the cryo-EM maps in this work.
Table S4. Validation statistics for the atomic models for R2TP-ΔNT_structure3 and R2TP-ΔNT_structure4.
Fig. S1. Cryo-EM of truncated human R2TP (R2TP-ΔNT).
Fig. S2. Resolution estimation for cryo-EM structures obtained from the subtracted particles.
Fig. S3. High-resolution details in the cryo-EM maps.
Legends for movies S1 and S2

Other Supplementary Material for this manuscript includes the following:

(available at advances.sciencemag.org/cgi/content/full/5/5/eaaw1616/DC1)

Movie S1 (.mov format). Several views of the multibody refinement strategy, showing the motions of the PIH1D1 region bound to a DII domain of RUVBL2, with respect to the rest of the molecule.
Movie S2 (.mp4 format). Conformational changes in the RUVBL2 subunit, from a closed conformation to an open ADP-filled conformation to an open ADP-empty conformation.

Table S1. Primers used for cloning in this work.

	**Primer 5'-3'**
RPAP3 400 Forward	GGTCATTGGGATGACGTGTTCCTG
RPAP3 665 reverse	TTAACCGCCGTAGCGTTTTTTCAGTTCTTCGAC

Table S2. Conditions used for cryo-EM data collection for all the cryo-EM maps in this work.

**Cryo-EM access codes**	(EMD-4552, EMD-4553, EMD-4554, EMD-4555, EMD-4556, EMD-4557)
**Data collection (for all structures)**	
Microscope	FEI Titan Krios
Detector	Gatan K2 (counting mode)
Calibrated magnification	130000
Voltage (kV)	300
Electron exposure (e–/Å^2)	47.5 (40 fractions)
Defocus range (μm)	-1.5 to -2.5
Pixel size (Å)	1.07

Table S3. Cryo-EM data refinement and validation statistics for all the cryo-EM maps in this work.

**Processing (R2TP-ΔNT_structure1)**	(EMD-4554)
Symmetry imposed	C1
Initial particle images (no.)	408781
Final particle images (no.)	136414
FSC threshold	0.143
Map resolution (Å)	3.97
Map resolution range (Å)	3.0-20.0
Map sharpening *B* factor (Å^2)	-150.95
**Processing (R2TP-ΔNT_structure2)**	(EMD-4555)
Symmetry imposed	C1

Initial particle images (no.)	408781
Final particle images (no.)	103427
FSC threshold	0.143
Map resolution (Å)	3.97
Map resolution range (Å)	3.0-20.0
Map sharpening *B* factor (Å^2)	-130.504
**Processing (R2TP-ΔNT_structure3)**	(EMD-4552, PDB ID 6QI8)
Symmetry imposed	C1
Initial particle images (no.)	408781
Final particle images (no.)	102100
FSC threshold	0.143
Map resolution (Å)	3.75
Map resolution range (Å)	3.0-10.0
Map sharpening *B* factor (Å^2)	-140.711
**Processing (R2TP-ΔNT_structure4)**	(EMD-4553, PDB ID 6QI9)
Symmetry imposed	C1
Initial particle images (no.)	408781
Final particle images (no.)	33466
FSC threshold	0.143
Map resolution (Å)	4.63
Map resolution range (Å)	3.0-10.0
Map sharpening *B* factor (Å^2)	-166.426
**Processing (R2TP-ΔNT_structure5)**	(EMD-4556)
Symmetry imposed	C1
Initial particle images (no.)	408781
Final particle images (no.)	102100
FSC threshold	0.143
Map resolution (Å)	3.97
Map resolution range (Å)	3.0-10.0
Map sharpening *B* factor (Å^2)	-147.472
**Processing (R2TP-ΔNT_structure6)**	(EMD-4557)
Symmetry imposed	C1
Initial particle images (no.)	408781
Final particle images (no.)	33466
FSC threshold	0.143
Map resolution (Å)	5.15

Map resolution range (Å)	3.0-10.0
Map sharpening *B* factor (Å^2)	-190.506

Table S4. Validation statistics for the atomic models for R2TP-ΔNT_structure3 and R2TP-ΔNT_structure4.

**Refinement R2TP-ΔNT_structure3**	(EMD-4552, PDB ID 6QI8)
Refinement software	phenix.real_space_refine
Initial model used (PDB code)	6FO1
Map sharpening *B* factor (Å^2)	-140.711
Model composition	
Non-hydrogen atoms	14789
Protein residues	1907
Ligands	6 ADP
B factors (Å^2)	
Average	39.69
R.m.s. deviations	
Bond lengths (Å)	0.004
Bond angles (°)	0.9
Validation	
MolProbity score	1.53
Clashscore	3.66
Poor rotamers (%)	0.13
Cbeta outliers (%)	0
Ramachandran plot	
Favored (%)	94.51
Allowed (%)	5.49
Disallowed (%)	0
Mask CC	0.82
**Refinement R2TP-ΔNT_structure4**	(EMD-4553, PDB ID 6QI9)
Refinement software	phenix.real_space_refine
Initial model used (PDB code)	6FO1
Map sharpening *B* factor (Å^2)	-166.426
Model composition	
Non-hydrogen atoms	14559
Protein residues	1879
Ligands	5 ADP
B factors (Å^2)	

Average	167.88
R.m.s. deviations	
Bond lengths (Å)	0.007
Bond angles (°)	1.082
Validation	
MolProbity score	2.05
Clashscore	10.33
Poor rotamers (%)	0.39
Cbeta outliers (%)	0
Ramachandran plot	
Favored (%)	91.01
Allowed (%)	8.99
Disallowed (%)	0
Mask CC	0.74

SUPPLEMENTARY FIGURES

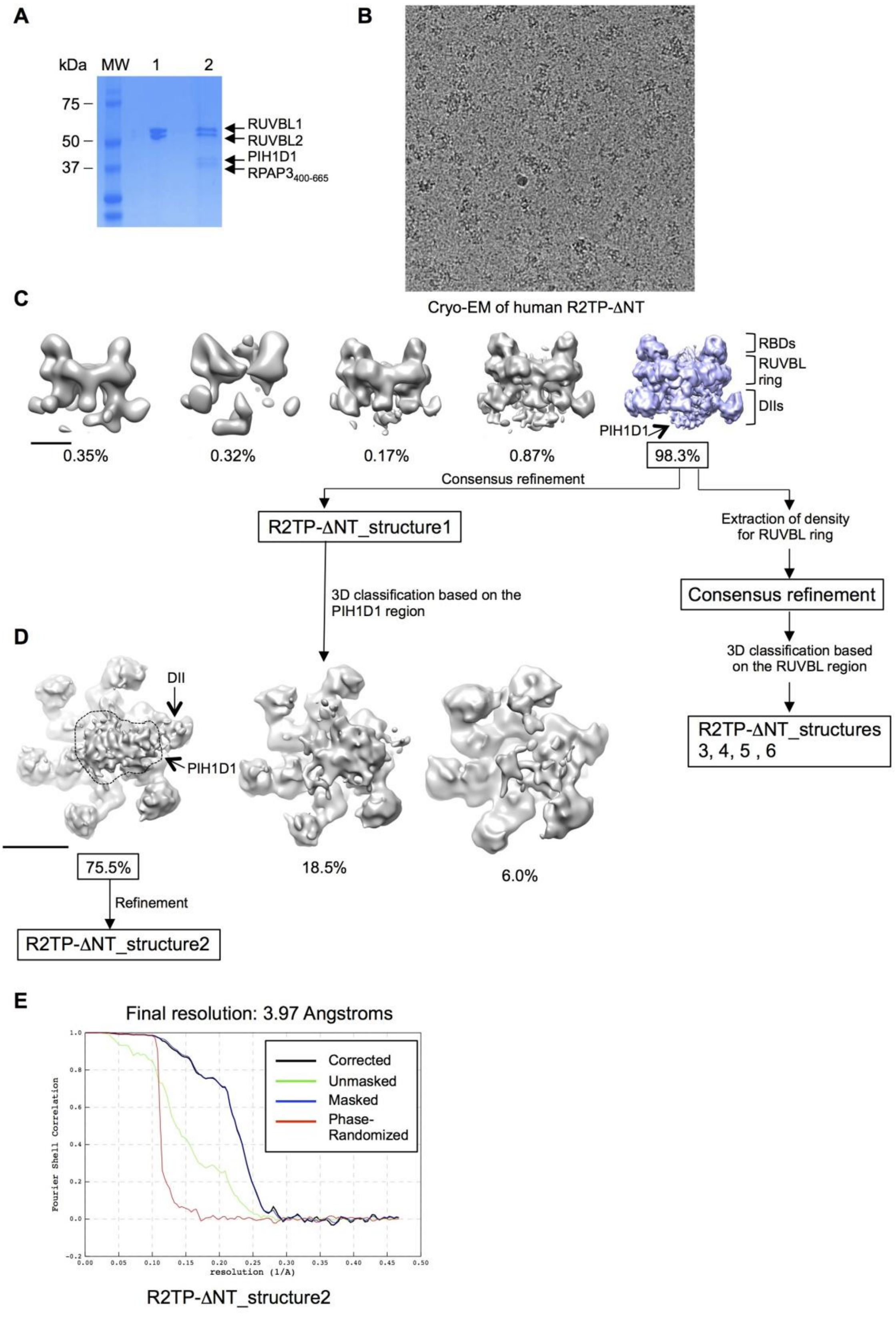

Fig. S1. Cryo-EM of truncated human R2TP (R2TP-ΔNT). (**A**) Strep-pull down experiment showing the interaction between RUVBL1-RUVBL2 and $RPAP3_{400\text{-}665}$-PIH1D1. MW stands for molecular weight markers. Lane 1 shows the RUVBL1-RUVBL2 complex containing a strep-tagged RUVBL2 subunit, only used for this experiment. The $RPAP3_{400\text{-}665}$-PIH1D1 complex is the same as in Fig. 1b. Lane 2 shows the material eluted from the beads. $RPAP3_{400\text{-}665}$-PIH1D1 binds to RUVBL1-RUVBL2. For cryo-EM, the ratio of $RPAP3_{400\text{-}665}$-PIH1D1 versus RUVBL1-RUVBL2 was increased to favour saturation of the RUVBL1-RUVBL2 complex. (**B**) Cryo-EM field of human R2TP-ΔTP. (**C**) 3D classification of the R2TP-ΔNT images into 5 subgroups. The most abundant class shows density for the RUVBL ring, the DII and RBD domains and PIH1D1. Scale bar represents 5.0 nm (**D**) Classification of the particles based on the variability of PIH1D1 into 3 subgroups. The most abundant group shows clear density for PIH1D1 and the displaced DII domain, and this was selected for further refinement. Scale bar represents 5 nm. (**E**) Resolution estimation of R2TP-ΔNT_structure2, using FSC and the gold standard as determined in RELION.

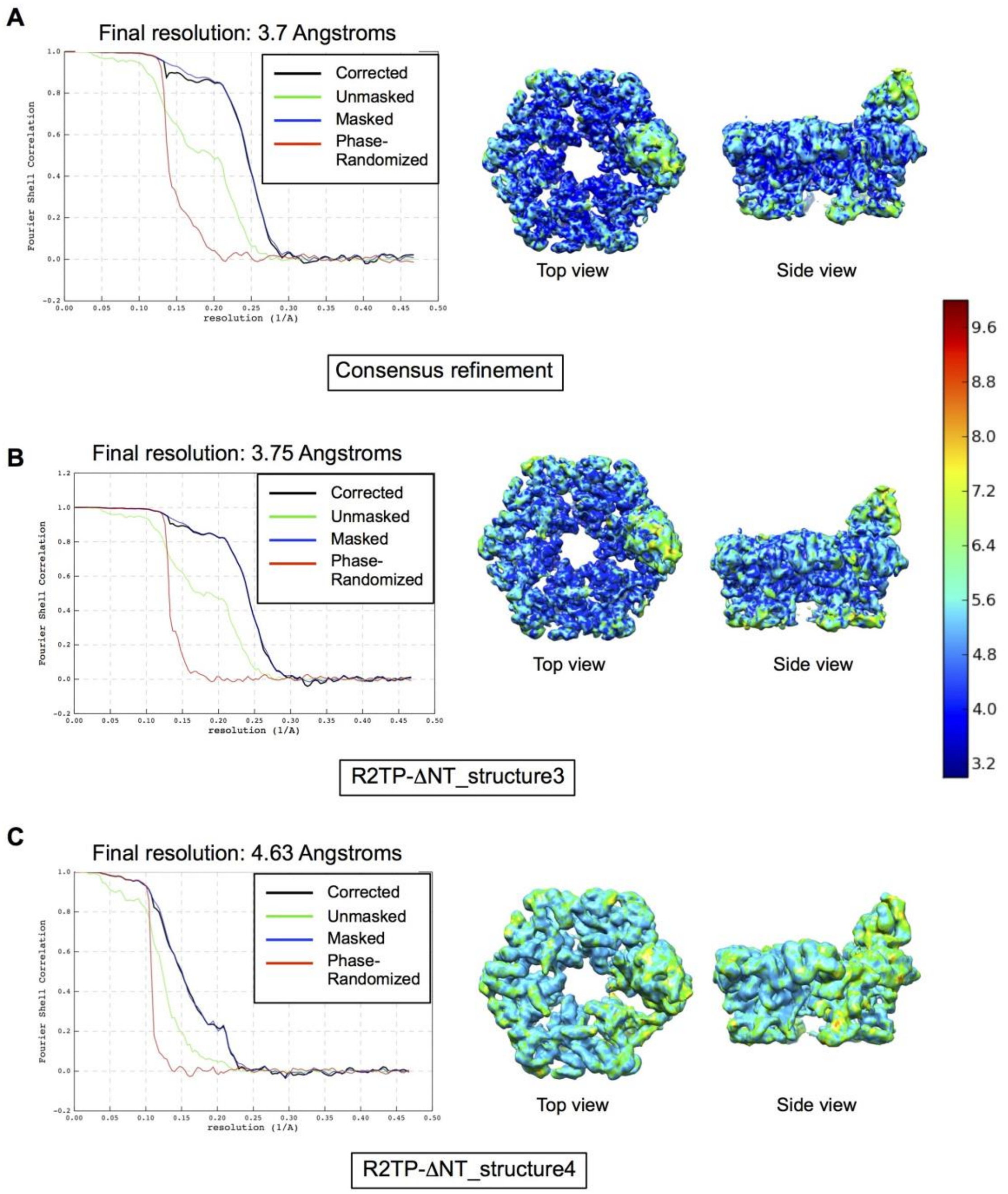

Fig. S2. Resolution estimation for cryo-EM structures obtained from the subtracted particles. (**A**) Resolution estimation of the structure obtained after the consensus refinement of all subtracted particles, using FSC and the gold standard as defined in RELION. Top and side views of the local resolution map are also shown. (**B**) As in “a” but for the ADP-filled conformation (R2TP-ΔNT_structure3). (**C**) As in “a” but for the ADP-empty conformation (R2TP-ΔNT_structure4).

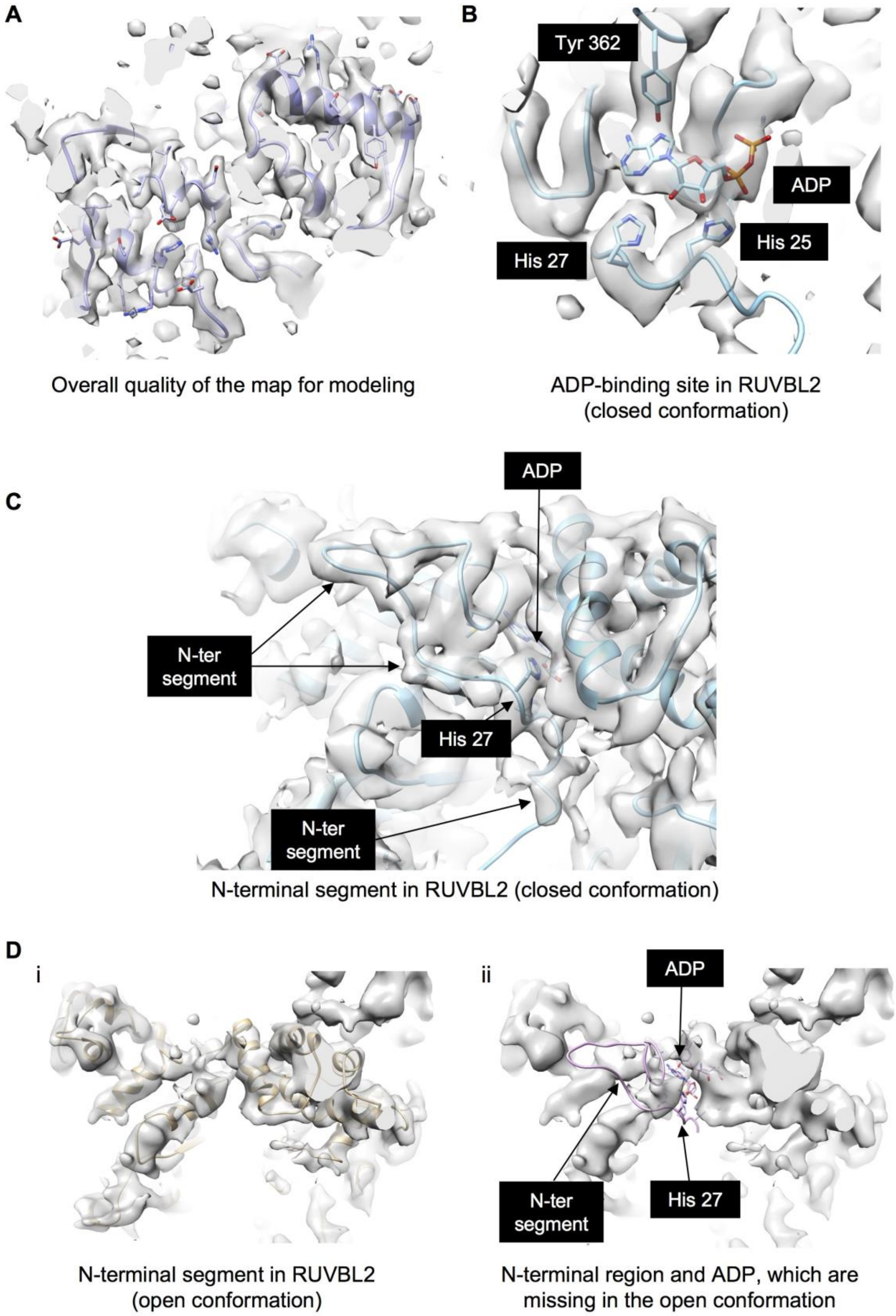

Fig. S3. High-resolution details in the cryo-EM maps. (**A**) View of a region in R2TP-ΔNT_structure3 where high-resolution details and density for side chains are clearly

observed. The cryo-EM density is shown as a transparent density where the atomic model is fitted in blue color. (**B**) Close-up view of the cryo-EM density and the model of the ADP-binding site of a RUVBL2 subunit in the closed conformation. Residues His 25, His 27 and Tyr 362 interacting with the ADP are labelled. (**C**) Close-up view of the cryo-EM density and the model of the N-terminal segment of a RUVBL2 subunit in the closed conformation. (**D**) (i). Close-up view of the cryo-EM density and the model of the ADP-binding site of a RUVBL2 subunit in the open conformation. Density for the nucleotide and the whole of the N-terminal segment is missing in the open conformation. (ii) To help comparison, the same region in the cryo-EM map shown in “i” but superimposed with the model for the N-terminal segment of RUVBL2 in the closed conformation (pink colour), to highlight that the open conformation does not show any density for the ADP or the N-terminal segment.

SUPPLEMENTARY MOVIES

Movie S1. **Several views of the multibody refinement strategy, showing the motions of the PIH1D1 region bound to a DII domain of RUVBL2, with respect to the rest of the molecule.** For this analysis, three “bodies” were defined, the RUVBL ring including the RBD domains and the internal part of the DII domains, the external segments of all DII domains except the one attached to PIH1D1, and the unit formed by PIH1D1 and the external part of the DII domain that it contacts.

Movie S2. **Conformational changes in the RUVBL2 subunit, from a closed conformation to an open ADP-filled conformation to an open ADP-empty conformation.** Only the regions conserved between the closed and ADP-empty conformation are shown, and thus, the N-terminal loop and the ADP are not shown. Movie starts from the closed conformation to the open ADP-filled and finally the ADP-empty conformation.
